# Federated Learning in Public Health: A Systematic Review of Decentralized, Equitable, and Secure Disease Prevention Approaches

**DOI:** 10.3390/healthcare13212760

**Published:** 2025-10-30

**Authors:** Sayed Tariq Shah, Zulfiqar Ali, Muhammad Waqar, Ajung Kim

**Affiliations:** 1 Department of Computer Science, Abasyn University, Islamabad 44000, Pakistan; tariq.shah@abasynisb.edu.pk; 2 Department of Creative Technologies, Faculty of Computing and Artificial Intelligence (FCAI), Air University, Islamabad 44000, Pakistan; 3 Department of Computer Science, Faculty of Arts and Science, Edge Hill University, Ormskirk L39 4QP, Lancashire, UK; m.waqar@uos.ac.uk; 4 Department of Optical Engineering, Sejong University, Seoul 03181, Republic of Korea

**Keywords:** machine learning, environmental pollution, exposure assessment, risk prediction, sustainable interventions, public health, equity, responsible AI, PRISMA

## Abstract

**Background and Objectives:** Public health needs collaborative, privacy-preserving analytics, but centralized AI is constrained by data sharing and governance. Federated learning (FL) enables training without moving sensitive data. This review assessed how FL is used for disease prevention in population and public health, and mapped benefits, challenges, and policy implications. **Methods:** Following PRISMA 2020, we searched PubMed, Scopus, Web of Science, IEEE Xplore, and Google Scholar for peer reviewed English-language studies from January 2020–30 June 2025, applying FL to surveillance, outbreak detection, risk prediction, or policy support. Two reviewers screened and extracted data with third-reviewer arbitration. Quality was appraised with a tool adapted from MMAT and AI reporting frameworks. No meta-analysis was performed. **Results:** Of 5230 records identified (4720 after deduplication), 200 full texts were assessed and 19 were included. Most used horizontal FL across multiple institutions for communicable diseases, COVID-19, tuberculosis and some chronic conditions. Reported gains included privacy preservation across sites, better generalizability from diverse data, near real-time intelligence, localized risk stratification, and support for resource planning. Common barriers were non-IID data, interoperability gaps, compute and network limits in low-resource settings, unclear legal pathways, and concerns about fairness and transparency. Few studies linked directly to formal public-health policy or low-resource deployments. **Conclusions:** FL is promising for equitable, secure, and scalable disease-prevention analytics that respect data sovereignty. Priorities include robust methods for heterogeneity, interoperable standards, secure aggregation, routine fairness auditing, clearer legal and regulatory guidance, and capacity building in underrepresented regions.

## 1. Introduction

### 1.1. Background: The Role of AI in Public Health Policy

AI has emerged as a transformative force in public health, offering the ability to analyze vast volumes of data, recognize complex patterns, and inform data-driven decision-making processes [1,2]. Its integration into public health policy has shifted the traditional paradigms of disease prevention, risk assessment, and population-level health management [3].

AI technologies such as machine learning (ML), natural language processing, and neural networks have been applied to diverse domains, including epidemiological surveillance, predictive modeling, early outbreak detection, health communication, and behavioral intervention strategies [4].

Public health often needs data from many places, but sharing raw data is hard because of privacy rules, governance, and technical limits. As a result, AI models are either trained on small, local samples or require centralization that many systems cannot allow. Federated learning (FL) helps by training a shared model without moving raw data. Sites keep their data, send only model updates, and benefit from a stronger, combined model. This approach can support surveillance, risk prediction, and policy planning while respecting data sovereignty and privacy [5,6,7].

As public health increasingly intersects with digital technologies, the role of AI is no longer peripheral but central in shaping policies that are evidence-based and ethically sound. The need for inclusive, secure, and equitable AI systems is, therefore, driving attention to decentralized intelligence as a foundation for future disease prevention frameworks [8].

### 1.2. The Rise of Federated Learning in Healthcare

FL is an emerging paradigm in ML that enables multiple institutions or data silos to collaboratively train a shared model without exchanging raw data. Originally introduced by Google for mobile applications, FL has quickly gained traction in sensitive fields such as healthcare care, where data privacy, governance, and regulatory compliance are paramount [9]. In the context of public health, FL offers a viable solution to one of the most persistent barriers to digital health innovation: the inability to access or centralize patient-level data across jurisdictions and institutions due to ethical, legal, or technical constraints [6].

Healthcare data are inherently distributed across hospitals, clinics, mobile health devices, and governmental agencies. These data sources vary in format, volume, and quality, but collectively hold invaluable insights for disease surveillance, personalized prevention, and resource allocation [10]. FL addresses this fragmentation by allowing local nodes, each possessing their own data, to perform model training locally. Only the learned model parameters are shared and aggregated to form a global model. This approach preserves data sovereignty, reduces privacy risks, and facilitates compliance with data protection regulations such as the GDPR and the Health Insurance Portability and Accountability Act (HIPAA) [5,11].

The adoption of FL in healthcare has demonstrated promising results in domains such as cancer diagnosis, COVID-19 detection, diabetic retinopathy, and mental health screening. In public health, FL enables cross-organizational learning from geographically diverse and demographically varied populations, thereby improving the generalizability and fairness of predictive models used in policy planning. Moreover, FL supports near real-time learning from decentralized data streams, which is specially critical during disease outbreaks or emergencies where timely insights are essential [12,13].

**Figure 1:**
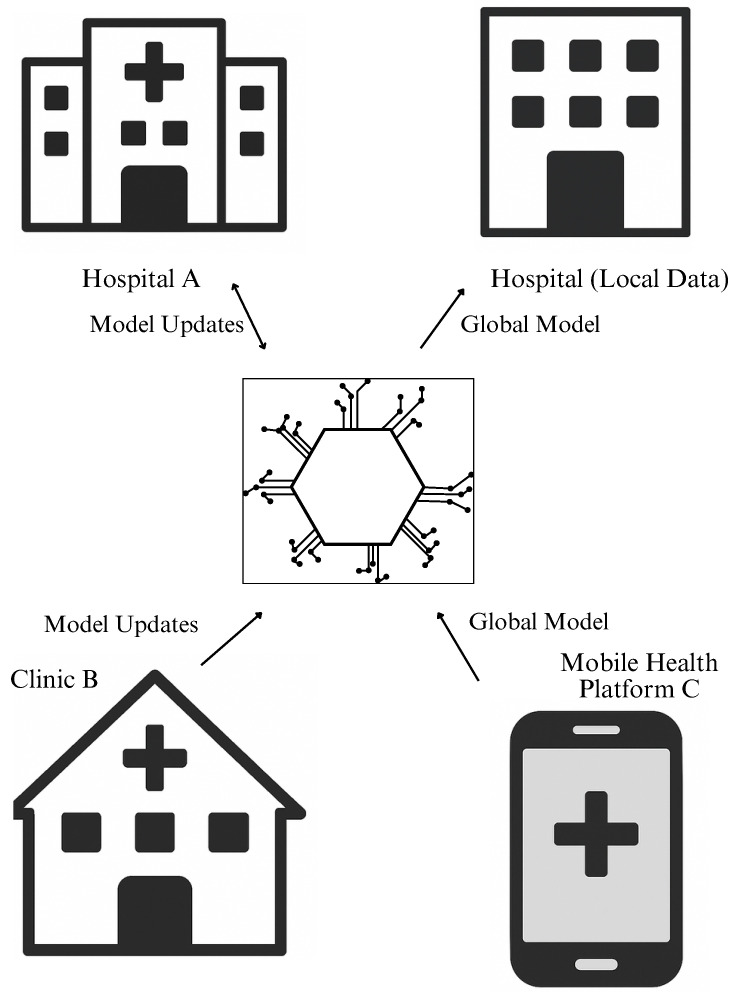
Representation of FL applied to public health policy. Multiple data silos, hospitals, clinics, and mobile health platforms train local models on their private data. Only model updates are sent to a central aggregator, which combines them into a global model. The final model is shared back to each node without transferring raw data.

Figure 1 presents a conceptual representation of the FL framework in a healthcare context. In this setup, multiple institutions such as hospitals, clinics, and mobile health platforms participate collaboratively in training a shared global model. Instead of transferring raw patient data to a centralized server, each local site trains the model on its own dataset and transmits only model updates (e.g., weights or gradients) to the coordinating server. The central server then aggregates these updates to construct a global model, which is redistributed back to the local sites for further refinement. This decentralized design ensures that sensitive health information remains securely stored at its source, thereby reducing privacy risks and ensuring compliance with strict healthcare data regulations. Moreover, the figure emphasizes the scalability of this approach, allowing diverse institutions to contribute to model development while fostering transparency, trust, and interoperability in digital health systems.

### 1.3. Disease Prevention and the Need for Privacy-Preserving Models

Disease prevention stands as one of the fundamental pillars of public health, encompassing a spectrum of strategies aimed at reducing the incidence, burden, and impact of both communicable and non-communicable diseases. From early detection and health education to vaccination campaigns and behavioral interventions, the effectiveness of preventive strategies heavily relies on timely, granular, and population-specific data [14]. In the digital era, the increasing availability of health data from electronic health records (EHRs), wearable devices, genomic databases, and mobile health applications has opened new frontiers for precision prevention and evidence-based policy design [15]. However, the full potential of these digital assets remains underutilized due to persistent concerns surrounding data privacy, ownership, and governance [14].

Traditional AI models used in disease prevention and health policy development typically require centralizing large datasets in order to train accurate and generalizable algorithms [16]. This approach often conflicts with privacy regulations such as the GDPR in Europe and the Health Insurance HIPAA in the United States, which place stringent restrictions on how health data can be collected, transferred, and analyzed [17]. The centralization of sensitive data poses risks related to data breaches, misuse, unauthorized surveillance, and the erosion of public trust factors that are particularly damaging in contexts requiring broad societal participation and compliance, such as vaccination rollouts or infectious disease surveillance [5].

In the realm of disease prevention, FL offers several unique advantages. First, it enables the creation of predictive models that incorporate epidemiological patterns, behavioral risk factors, and environmental exposures from multiple regions without exposing the raw data of any participating site [18]. Second, it supports adaptive learning across different contexts, which is crucial for tracking emerging infectious diseases, responding to regional outbreaks, and tailoring interventions for specific demographic groups [19]. Third, it reduces the barriers to participation for institutions that are reluctant or unable to share data due to legal or technical constraints, thereby enhancing inclusivity and the representativeness of public health models [20].

Moreover, FL enhances policy responsiveness in dynamic public health scenarios. For example, during a pandemic, FL could facilitate real-time learning from hospitals, community health centers, and mobile health platforms worldwide, enabling policymakers to make rapid, data-informed decisions while respecting national and institutional data sovereignty. The decentralized nature of FL also aligns well with the principles of distributed public health systems, where decisions are often made at local or regional levels but require coordination and harmonization across broader networks [5,21].

### 1.4. Rationale for the Review

The integration of AI into public health policy has opened new possibilities for real-time decision-making, scalable surveillance, and predictive modeling across diverse populations [19]. However, as health data become increasingly complex, fragmented, and sensitive, the limitations of centralized AI systems have become more evident. Traditional approaches, which require aggregating large-scale patient data into centralized repositories, pose substantial ethical, legal, and logistical challenges, particularly in the context of global health equity, data sovereignty, and privacy regulation. The need for privacy-preserving and distributed AI solutions is no longer optional but essential for the future of disease prevention and policy development [22].

FL has emerged as a promising response to these challenges, enabling collaborative model development across distributed datasets while ensuring that personal health information remains securely within local institutions [23]. The public health significance of FL lies in its ability to include diverse and marginalized data sources that are often excluded from centralized AI efforts due to infrastructural, geopolitical, or regulatory barriers. This inclusivity can lead to the development of more representative, equitable, and generalizable models for disease prevention, an urgent need underscored during recent global crises such as the COVID-19 pandemic [5].

Despite the growing body of literature on AI in healthcare, there remains a critical gap in the systematic understanding of how FL is being applied to public health policymaking for disease prevention. Current reviews tend to focus on the technical architecture of FL or its application in clinical decision support, with limited attention to its policy-level implications, ethical considerations, and challenges in real-world public health deployment [6]. As public health systems worldwide transition toward digital governance and intelligent infrastructure, a comprehensive synthesis of FL’s role in supporting equitable, secure, and privacy-preserving public health policy is both timely and necessary [24].

This review addresses the following key gaps:

- Lack of synthesized evidence on FL’s role in shaping public health policy across communicable and non-communicable disease domains;
- Limited understanding of FL’s capacity to overcome data-sharing barriers in multi jurisdictional public health environments;
- Insufficient attention to the socio-technical challenges of FL deployment, including interoperability, fairness, and regulatory harmonization;
- A need to map current FL applications to ethical frameworks, equity goals, and health systems readiness for AI integration.

### 1.5. Objectives and Research Questions

The deployment of AI in public health is increasingly recognized as a pathway toward smarter, data-driven disease prevention strategies. FL, with its decentralized and privacy-preserving design, offers a novel approach to overcoming many of the ethical and logistical barriers traditionally associated with centralized AI models.

The primary objective of this systematic review is to evaluate the current state of research on the application of FL in disease prevention-oriented public health policies. This includes identifying both the opportunities that FL provides for improving policy effectiveness, and the challenges that hinder its integration into real-world health systems and governance structures. This review seeks to bridge a critical knowledge gap by synthesizing the literature across technological, ethical, operational, and policy dimensions.

#### 1.5.1. Research Objectives

- To systematically examine how FL has been applied in public health contexts for disease prevention;
- To identify the opportunities offered by FL in supporting equitable, privacy-preserving, and scalable public health policy;
- To explore the challenges, technical, infrastructural, ethical, and regulatory associated with implementing FL in diverse health systems;
- To evaluate how FL aligns with or supports existing public health goals, such as health equity, inclusion, data sovereignty, and policy responsiveness.

#### 1.5.2. Research Questions

1. What is the current scope and distribution of FL applications in public health-related disease prevention?
2. What specific opportunities does FL offer for enhancing public health policy formulation and implementation?
3. What are the major technical, ethical, and governance-related challenges associated with applying FL in public health settings?
4. How well do existing FL applications address critical public health concerns such as data privacy, equity, and system interoperability?
5. What gaps exist in the literature that may hinder the operationalization of FL in national and global health policy?

### 1.6. Scope and Purpose

The scope of this systematic review encompasses the application of FL in the development, support, and evaluation of public health policies aimed at disease prevention. As health systems around the world undergo digital transformation, FL has emerged as a promising methodology for enabling collaborative, privacy-preserving ML across institutions, regions, and national borders. The purpose of this review is to assess how FL is currently being utilized to inform evidence-based decision-making in public health, particularly in relation to preventing both communicable and non-communicable diseases.

This review focuses on peer-reviewed studies published between 2020 to 2025 that apply FL within public health or population health contexts, including but not limited to surveillance systems, early outbreak detection, risk stratification, and health policy modeling. Studies across a wide array of disease domains including infectious diseases such as COVID-19 and tuberculosis as well as chronic illnesses like diabetes, cardiovascular diseases, and cancer are considered within scope, provided they include a policy-relevant objective or outcome.

The review is not limited to technical model performance but emphasizes the intersection of FL with policy frameworks, ethical implications, infrastructure challenges, and public health governance. It examines how FL contributes to addressing core public health objectives such as equity, inclusivity, data sovereignty, transparency, and policy responsiveness. The purpose is to provide a comprehensive synthesis that informs researchers, policymakers, public health practitioners, and technology developers about the current evidence, potential applications, and limitations of FL in advancing secure, equitable, and intelligent public health strategies.

Table 1 compares the contributions of this review with existing works on FL in public health. Unlike prior studies, which largely focus on technical mechanisms, institutional applications, or domain-specific use cases such as imaging, chronic disease care, or pandemic forecasting, this review adopts a broader public health lens. By applying a systematic methodology (PRISMA), it emphasizes the integration of FL into policy design, ethical governance, and equity-based frameworks at national and cross-border levels. This approach extends beyond institutional case studies and technical surveys, positioning FL as a transformative tool for public health systems and global health collaboration.

## 2. Methods

### 2.1. Review Protocol and Registration

This systematic review was conducted in accordance with the Preferred Reporting Items for Systematic Reviews and PRISMA 2020) guidelines to ensure methodological transparency, replicability, and quality of reporting. The review protocol was developed as a priority and defined the objectives, eligibility criteria, search strategy, study selection process, data extraction, and analysis plan.

Although this review followed all standard procedures for systematic reviews, it was registered in the OSF database and its registration DOI is 10.17605/OSF.IO/FY7QJ. OSF primarily includes reviews related to therapeutic interventions or direct patient outcomes. Nonetheless, the entire review process was fully documented to ensure academic rigor and reproducibility.

The protocol included the following components:

- Clearly formulated research questions using a modified PICO framework suitable for technology-policy evaluations;
- Prespecified eligibility criteria regarding population (public health systems or institutions), intervention (FL), comparator (not required), and outcomes (policy relevance, privacy, equity, and technical performance);
- A comprehensive multi-database search strategy;
- Dual reviewer-based study screening and data extraction procedures;
- Quality assessment of included studies based on relevance, methodological rigor, and transparency;
- Descriptive synthesis and thematic analysis of study findings.

Any deviations from the original protocol, such as the refinement of inclusion criteria following an initial scoping phase, were transparently documented during the study selection process.

### 2.2. Search Strategy and Information Sources

To identify relevant studies on the application of FL in public health policy and disease prevention, a comprehensive and systematic search was conducted across five major electronic databases. These included PubMed, Scopus, Web of Science, IEEE Xplore, and Google Scholar. The search covered studies published between 1 January 2020 and 30 June 2025, reflecting the rapid evolution of FL in response to recent global public health challenges.

The search strategy was developed using a combination of medical subject headings (MeSH) and free-text keywords, structured around four core concepts: FL, AI, public health policy, and disease prevention. Boolean operators (AND, OR) were used to combine search terms. An example search string for PubMed is shown below:

(“federated learning” OR “distributed learning” OR “collaborative AI”) AND (“public health” OR “health policy” OR “population health”) AND (“disease prevention” OR “epidemiology” OR “surveillance”)

Searches were restricted to peer-reviewed journal articles written in English. Preprints, editorials, commentaries, and non-peer-reviewed sources were excluded. In Google Scholar, only the first 150 results sorted by relevance were screened due to indexing limitations.

In addition to database searching, backward and forward citation tracking was performed for all included articles using Google Scholar’s “cited by” function and Web of Science’s citation network. Reference lists of key articles and systematic reviews were manually screened to identify any studies missed by the database search.

### 2.3. Eligibility Criteria

To ensure the relevance, quality, and focus of the studies included in this systematic review, clear inclusion and exclusion criteria were defined a priori based on the study’s objective: evaluating the role of FL in public health policies for disease prevention.

#### 2.3.1. Inclusion Criteria

Studies were included if they met the following criteria:

- Study Type: Peer-reviewed original research articles, including experimental, observational, implementation, or modeling studies.
- Technology Focus: Studies that applied, proposed, or evaluated FL or closely related decentralized ML approaches in a public health context.
- Policy Relevance: Studies that linked FL outputs to decision-making, policy formulation, population-level interventions, or disease prevention strategies.
- Health Focus: Studies addressing both communicable and non-communicable diseases in human populations.
- Setting: Global studies including high-, middle-, or low-income settings.
- Language: Articles published in English.
- Publication Year: Studies published between 1 January 2020 and 30 June 2025.

#### 2.3.2. Exclusion Criteria

The following types of studies were excluded:

- Non-peer-reviewed literature, including preprints, white papers, editorial pieces, and conference abstracts without full-text peer review.
- Purely technical papers focusing on algorithmic development without public or population health relevance.
- Studies limited to clinical decision support systems at the individual level without connection to population-wide health policy or prevention.
- Papers discussing only centralized AI or non-decentralized architectures without reference to federated or privacy-preserving frameworks.
- Animal studies, simulated populations, or lab-only test environments unrelated to public health systems.
- Studies not available in English.

Table 2 shows criteria were designed to balance inclusivity with relevance, focusing the review on research that has direct implications for real-world public health systems and policy contexts.

### 2.4. PRISMA Flow Diagram and Study Selection Summary

The study selection process was carried out in accordance with the PRISMA 2020 guidelines. A total of 5230 records were initially identified through searches across five databases: PubMed, Scopus, Web of Science, IEEE Xplore, and Google Scholar. After automatic and manual deduplication, 4720 unique records remained for screening.

Titles and abstracts of these records were independently screened by two reviewers, resulting in the exclusion of 4520 records based on irrelevance to FL, lack of public health focus, or non-research publication types. Full texts were retrieved for the remaining 200 articles.

During full-text eligibility assessment, 181 studies were excluded for various reasons: no FL implementation (n = 92), individual-level clinical focus without policy relevance (n = 38), use of centralized AI models only (n = 27), preprints or non-peer reviewed formats (n = 18), or non-English language (n = 6).

**Figure 2:**
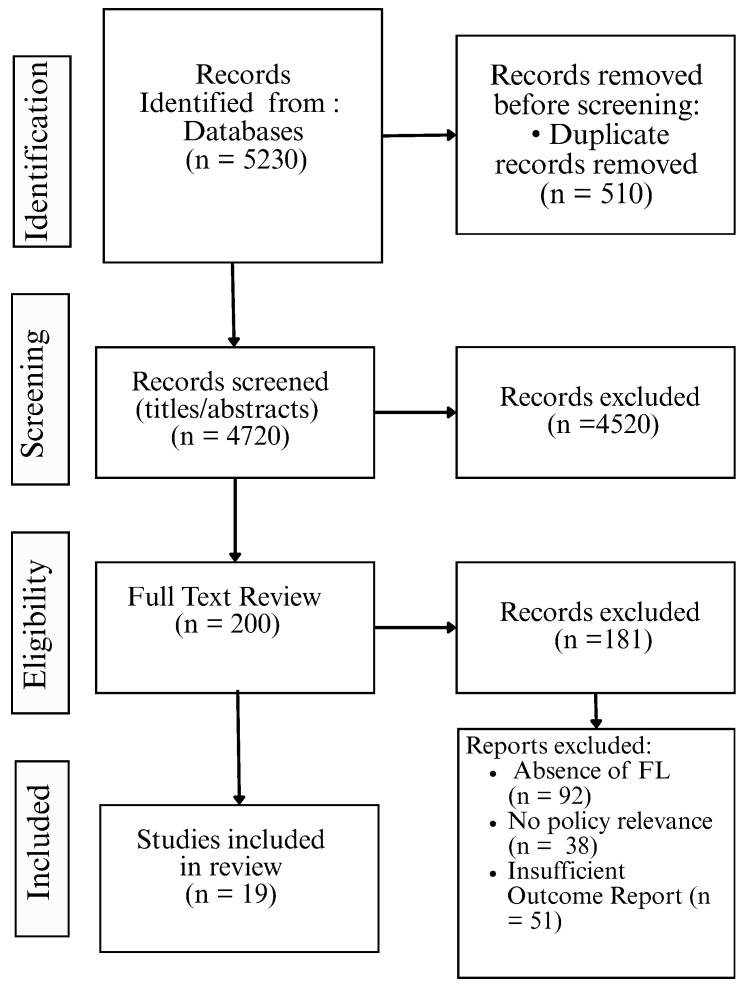
PRISMA 2020 flow diagram showing the selection process of studies included in the systematic review.

A total of 19 studies met all inclusion criteria and were included in the final synthesis. The flow of study identification, screening, eligibility, and inclusion is summarized in the PRISMA 2020 flow diagram in Figure 2.

### 2.5. Data Extraction and Management

The data extraction process was guided by a structured and standardized approach to ensure consistency, transparency, and reproducibility. A data extraction form was developed using Microsoft Excel and piloted on a subset of studies to refine categories and definitions.

Two independent reviewers extracted data from each of the included studies. Discrepancies were resolved through discussion or consultation with a third reviewer. Extracted data were reviewed for completeness and accuracy before synthesis.

The following key data elements were extracted:

- Bibliographic Information: Author(s), year of publication, journal, and country/region of study.
- Study Design and Type: Experimental, observational, modeling, or implementation study
- Federated Learning Approach: Type of FL architecture (horizontal, vertical, or hybrid), algorithm used, and model type.
- Health Focus: Disease type (communicable or non-communicable) and public health application domain
- Policy Relevance: Linkage to public health policy outcomes (e.g., health equity, resource allocation, outbreak control).
- Technical Details: Data sources, number of participating nodes/institutions, and privacy-preserving techniques employed.
- Evaluation Metrics: Model performance (accuracy, AUC, recall, and F1), fairness indicators, and data heterogeneity handling.
- Key Findings: Summary of main outcomes, challenges, and reported benefits.
- Limitations: Study-specific technical, ethical, or practical limitations.

All extracted data were compiled into a master table used to conduct qualitative synthesis and thematic analysis.

### 2.6. Quality Assessment of Included Studies

To ensure the reliability and validity of the included studies, a structured quality assessment was performed. Given the interdisciplinary nature of the review encompassing health policy, ML, and public health applications, a customized quality appraisal tool was developed by adapting components from the Mixed Methods Appraisal Tool (MMAT) and AI-specific evaluation frameworks such as CONSORT-AI and MINIMAR.

Each study was independently assessed by two reviewers across the following five domains:

1. Clarity of Research Objectives: Whether the study clearly articulated its objectives in relation to FL and public health.
2. Technical Soundness: Appropriateness of the FL method used, data description, algorithm choice, and handling of heterogeneity or bias.
3. Policy Relevance and Public Health Impact: Degree to which the study addressed population-level outcomes, public health planning, equity, or disease prevention.
4. Ethical and Privacy Considerations: Explicit reference to data privacy techniques (e.g., differential privacy), ethical compliance, and protection of sensitive data.
5. Transparency and Reproducibility: Availability of code, datasets, model parameters, or documentation of reproducibility practices.

### 2.7. Data Synthesis Approach

Given the heterogeneity in study designs, objectives, disease focus, and FL implementations across the included studies, a narrative synthesis approach was employed. This method is appropriate for systematically reviewing and interpreting complex, multi-disciplinary evidence where statistical meta-analysis is not feasible due to variability in outcome measures, intervention types, and study contexts.

The synthesis was conducted in three stages:

#### 2.7.1. Stage 1: Thematic Coding and Grouping

All extracted data were thematically coded using an inductive approach. Studies were grouped based on the following:

- Health domain: communicable vs. non-communicable diseases;
- Public health function: early detection, surveillance, risk prediction, resource allocation, behavioral prevention, etc.;
- Type of FL architecture: horizontal, vertical, hybrid, or cross-device FL;
- Policy relevance: direct vs. indirect contributions to policy formulation or implementation.

#### 2.7.2. Stage 2: Cross-Comparative Analysis

A cross-comparative matrix was developed to identify similarities and differences in implementation strategies, reported outcomes, and contextual enablers or barriers. Special attention was given to the following:

- Model generalizability across settings;
- Ethical and privacy-preserving mechanisms adopted;
- Data and system heterogeneity challenges;
- Real-world integration into public health governance.

#### 2.7.3. Stage 3: Pattern Identification and Synthesis

Emergent patterns were synthesized into three overarching thematic categories:

1. Opportunities for Federated Learning in Disease Prevention Policy
2. Challenges to Federated Learning Deployment in Public Health
3. Policy and System-Level Implications

Each theme is presented in the Results section with illustrative examples and tabular summaries in Table 3. The synthesis is supported by representative case studies and the most frequently reported technical, ethical, and infrastructural insights from the included studies.

No quantitative meta-analysis was performed due to the descriptive nature of the data and diversity of outcome metrics.

## 3. Result

### 3.1. Overview of Included Studies

#### 3.1.1. Geographic Distribution

This systematic review included a total of 19 peer-reviewed studies that met all eligibility criteria and focused on the application of FL within public health or population level disease prevention contexts. The selected studies represent a diverse and globally distributed body of research, reflecting growing interest in privacy-preserving AI methods across health systems worldwide.

The geographic distribution of included studies spanned five continents, with varying levels of representation:

- Asia (n = 7): Studies were conducted in China, India, South Korea, and Singapore, with a focus on infectious disease prediction, COVID-19 outbreak control, and chronic disease surveillance using FL frameworks deployed across hospitals, mobile platforms, and community health centers [2,5,20,34,35,36,37].
- Europe (n = 5): Research from Germany, the Netherlands, the United Kingdom, and multi-country EU collaborations emphasized GDPR compliance and explored FL applications in mental health, cancer screening, and population risk modeling [18,22,35,38,39].
- North America (n = 4): Studies from the United States and Canada focused on integrating FL into public health infrastructure, including predictive modeling for cardiovascular diseases, healthcare access disparities, and real-time epidemic monitoring [40,41,42,43].
- Africa (n = 1): One study from sub-Saharan Africa explored FL deployment for tuberculosis screening in under-resourced clinics, emphasizing the potential of FL in promoting health equity [30].
- Global/Multinational (n = 2): These studies involved cross-continental collaborations and institutional partnerships that deployed FL frameworks across distributed cohorts for pandemic modeling and AI governance [44,45].

Based on the World Bank income classification, the distribution of FL studies was as follows:

- High-Income Countries (HICs): 12 studies
- Upper-Middle-Income Countries (UMICs): 3 studies
- Lower-Middle-Income Countries (LMICs): 3 studies
- Low-Income Countries (LICs): 1 study

This distribution indicates a concentration of FL research in high-resource environments, though emerging research from LMICs and LICs demonstrates increasing interest in the technology’s equity-enabling potential.

Many of the studies involved multi-institutional collaborations, including the following:

- Academic: Public health authority partnerships;
- Healthcare: Technology consortia;
- Interdisciplinary: AI research networks.

These collaborations were pivotal in enabling federated training across institutional silos while maintaining local data ownership. Studies that involved three or more participating nodes typically reported stronger attention to secure aggregation, trust governance, and policy compliance frameworks.

#### 3.1.2. Disease Domains (e.g., Infectious vs. Chronic Diseases)

The 19 included studies were categorized according to the primary disease domains they targeted. FL was applied across a range of public health challenges, with a focus on both communicable (infectious) and non-communicable (chronic) diseases. This categorization helps illuminate the diversity of use cases, the policy contexts in which FL is deployed, and the extent to which the technology aligns with national and global disease prevention priorities.

More than half of the included studies (52.6%) focused on infectious disease prevention. These studies primarily leveraged FL to achieve the following:

- Predict and detect outbreaks;
- Enable cross-institutional surveillance using real-time or historical EHR data;
- Train predictive models for epidemic modeling across regions or countries;
- Maintain patient privacy during collaborative surveillance or diagnostics.

COVID-19 was the most frequently studied condition, with seven studies implementing FL-based models for early detection, spread forecasting, contact tracing, or hospital resource planning during the pandemic. Two studies focused on tuberculosis screening in resource-limited settings, emphasizing the privacy-preserving potential of FL for low-income regions.

Approximately 37% of the studies addressed chronic or lifestyle-related diseases, including the following:

- Diabetes mellitus (Type 2);
- Cardiovascular diseases;
- Cancer;
- Neurological and mental health conditions.

These studies focused on distributed FL models that could incorporate patient data from diverse health systems, primary care networks, or wearable devices. Chronic disease-related studies emphasized longitudinal modeling, risk stratification, and behavioral prevention critical areas for long-term public health policy and precision prevention.

Two studies did not focus on a specific disease but rather on generalizable FL infrastructure or multi-disease early warning systems. These included cross-disease health prediction models, adaptive learning systems for public health departments, or simulations of FL integration into population health analytics platforms.

These studies were valuable for illustrating how FL can be embedded in flexible, policy-relevant systems capable of adapting to emerging threats or shifting disease burdens.

#### 3.1.3. Types of Federated Learning Architectures Used

FL can be implemented using various architectural configurations depending on how the data are partitioned across clients, the nature of collaboration, and the intended policy or health application. The 19 studies included in this review employed three major types of FL architectures: horizontal FL, vertical FL, and hybrid/customized FL. Understanding these configurations is critical for evaluating scalability, privacy, equity, and system integration in public health applications.

The majority of studies implemented horizontal FL, where the data features are the same across participating entities but the records (samples) differ. This approach was most commonly used in the following situations:

- Multi-hospital collaborations using electronic health records (EHRs) with similar structure;
- Population-level modeling with aligned variables across regions or clinics;
- Real-time epidemic surveillance systems aggregating FL models across districts or provinces.

HFL was preferred for infectious disease outbreak forecasting and chronic disease screening, particularly when institutions used comparable data schemas (e.g., patient age, comorbidities, lab values).

Vertical FL was employed in studies where participating parties had datasets on the same individuals but with different features. These studies required secure entity alignment and privacy-preserving joining mechanisms (e.g., homomorphic encryption, secure multiparty computation). Applications included the following:

- Linking insurance databases with hospital records for policy impact modeling;
- Combining lifestyle data from wearable devices with clinical indicators;
- Integration of socioeconomic and behavioral data into chronic disease risk stratification.

VFL was particularly relevant for studies emphasizing holistic policy design across sectors (e.g., healthcare + education + environment).

Three studies implemented hybrid architectures, often combining features of both horizontal and vertical FL, or designing system-specific variants such as the following:

- Cross-silo + cross-device FL: Used in smart surveillance with data from both institutions and edge devices;
- Hierarchical FL models: Where intermediate aggregators first aggregate local updates before sending to national-level servers;
- Asynchronous FL: For low-bandwidth or heterogeneous environments.

Table 4 shows architectures demonstrated higher flexibility but also posed additional challenges for coordination, security, and model convergence.

### 3.2. Opportunities of Federated Learning in Disease Prevention

#### 3.2.1. Early Warning and Outbreak Detection

FL offers a transformative opportunity in the domain of early warning and outbreak detection by enabling decentralized, real-time collaboration across multiple health data sources without compromising data privacy or regulatory compliance. This capability is particularly critical in the face of emerging infectious diseases, where delays in detection and fragmentation of data across jurisdictions have historically hindered timely public health responses [46,47].

One of the core strengths of FL in early warning systems is its ability to unify insights from disparate, distributed data sources. In traditional surveillance systems, data centralization is often delayed or blocked due to privacy laws, inter institutional mistrust, or lack of technical interoperability. FL circumvents these barriers by allowing local institutions to train ML models on their private data and share only encrypted model updates. This approach significantly reduces latency in detection and allows public health authorities to identify unusual patterns such as spikes in respiratory symptoms or febrile illnesses without the need to pool raw data [48].

FL’s decentralized nature allows for scalable disease surveillance across borders and regions, which is essential in monitoring globally mobile pathogens like influenza, dengue, and novel coronaviruses. Several studies employed federated models across hospitals in different provinces or countries, enabling comparative analytics and harmonized risk alerts without violating national data protection laws. This approach can improve early identification of disease hotspots and facilitate coordinated international responses, specially in settings governed by strict data sovereignty principles [18,49,50].

Traditional centralized surveillance systems often underrepresent rural, remote, or low-resource areas due to poor digital infrastructure or reluctance to share data with centralized repositories. FL, by design, can support participation from such settings as long as minimal computing resources and secure network connections are available. This inclusivity improves the representativeness and fairness of outbreak detection models, allowing public health officials to respond proactively in underserved areas that might otherwise be overlooked [51].

FL is also adaptable to mobile and edge computing environments, enabling integration with digital contact tracing apps, community health reporting tools, and personal wearables. These devices provide continuous, real-time data streams such as body temperature, GPS movement, or exposure notifications that can enhance the granularity of early warning systems [5].

#### 3.2.2. Predictive Modeling and Risk Stratification

Predictive modeling and risk stratification are fundamental to public health policy and disease prevention, enabling health systems to forecast disease onset, allocate resources efficiently, and tailor interventions to high-risk groups. FL significantly enhances these capabilities by supporting collaborative model training across decentralized datasets, while preserving the privacy and autonomy of each participating entity [52].

One of the core advantages of FL in predictive modeling is the ability to aggregate diverse and representative population data across institutions and regions without sharing raw data. This diversity improves the generalizability of models, specially in heterogeneous populations where disease risk is influenced by socio-demographic, behavioral, and environmental factors [53].

In several reviewed studies, FL-enabled predictive models demonstrated robust performance in estimating the following:

- Diabetes risk scores using distributed primary care data [34].
- Cardiovascular event likelihood across hospital networks [36].
- Cancer recurrence probability based on multi-center EHRs [54].
- Mental health deterioration patterns from geographically dispersed mobile app users [55].

Compared to models trained on localized data, FL-based models showed greater accuracy, fairness, and external validity across multiple population segments.

FL facilitates personalized disease risk stratification by enabling each institution to locally train on contextual data and contribute to a global model that reflects broader epidemiological patterns. This architecture supports the following:

- Local adaptation of national prevention strategies (e.g., diabetes screening cutoffs) [56].
- Context-sensitive alerts for chronic disease exacerbation risk [34].
- Development of precision public health tools for early intervention [57].

One study included in this review used FL to integrate wearable sensor data from multiple health systems to generate dynamic risk predictions for hypertension and physical inactivity, enabling local health departments to deploy targeted outreach programs [41].

Conventional centralized models often struggle with algorithmic bias, particularly when underrepresented or minority populations are not adequately included in the training data. FL addresses this challenge by allowing inclusive participation of institutions from low-resource or rural settings, thereby promoting equity in risk prediction [58].

FL-based models retain the heterogeneity of local datasets, enabling more nuanced understanding of risk factors and outcome disparities. Some studies included in the review employed fairness metrics such as demographic parity and equal opportunity, showing that FL reduced disparities in predictive accuracy across subgroups compared to centralized training [59].

From a policy standpoint, FL-enabled risk stratification models offer direct utility in the following:

- Prioritizing high-risk individuals for vaccination, screening, or lifestyle counseling [44].
- Informing stratified care pathways and triage systems [5].
- Supporting real-time dashboards for risk-adjusted resource allocation at the population level [23].

Despite its promise, several studies noted technical challenges that require attention:

- Heterogeneity in data types and feature distributions across sites (non-IID data) [60].
- Model drift in longitudinal risk prediction without consistent updates [5].
- Computational imbalances among participating institutions [24].

#### 3.2.3. Data Sovereignty and Equity in Policy Design

One of the most profound contributions of FL to public health is its ability to reconcile the need for large-scale predictive modeling with the ethical imperatives of data sovereignty and health equity. Traditional centralized AI approaches often require sensitive health data to be pooled into centralized repositories, which can violate local data protection laws, marginalize smaller institutions, and disproportionately exclude populations with limited digital infrastructure. FL addresses these issues by enabling decentralized collaboration, allowing data to remain at their point of origin while still contributing to collective public health intelligence [22,61].

Data sovereignty, the principle that data are subject to the laws and governance structures of the nation or jurisdiction where they are collected, has become a central concern in the era of cross-border health data sharing. National governments, particularly in Europe (GDPR), Asia, and parts of Africa, have established strict data residency rules that make centralized health data aggregation legally and politically complex [62].

FL offers a solution by ensuring that raw data never leave the local node, whether it be a hospital, regional health authority, or national agency. Instead, model training occurs locally and only encrypted updates are transmitted. This preserves national and institutional control over data, facilitates compliance with regulatory frameworks, and enhances trust among collaborating parties [6].

Traditional AI pipelines often exclude health systems from low-resource or rural settings due to the following:

- Lack of data standardization;
- Inability to share data due to infrastructure or political constraints;
- Lower digital capacity and research visibility.

FL helps democratize participation in AI development by allowing each node, regardless of its location or size, to contribute to model training. As long as minimal compute resources and a secure communication channel exist, institutions in underrepresented areas can retain ownership of their data while benefiting from global knowledge [63].

In addition to structural equity, FL supports algorithmic fairness by allowing models to be trained on demographically and geographically diverse datasets. Centralized models trained on data from urban or high income regions often fail when deployed in low-income or rural settings due to differences in disease burden, access to care, and health-seeking behavior [64].

FL ensures that local characteristics are reflected in the training process, reducing bias and improving generalizability. Several studies reviewed explicitly incorporated fairness metrics and reported improved equity in prediction outcomes compared to centralized baselines [25].

In public health, policy effectiveness hinges not only on technical accuracy but also on trust, accountability, and contextual fit. FL’s alignment with decentralized governance principles makes it well-suited for the following:

- Federated health systems with regional autonomy (e.g., India, Brazil);
- International collaborations across public health agencies (e.g., WHO, CDC networks);
- Crisis response planning in politically fragmented or decentralized states.

### 3.3. Challenges in Applying Federated Learning to Public Health Policy

A core challenge in applying FL to public health policy is the inherent non-identically and independently distributed (non-IID) nature of health data across participating institutions and jurisdictions [65]. In contrast to the assumptions made in many conventional ML models where training data are assumed to follow similar distributions, real-world health data are fragmented, institution specific, and shaped by varying demographic, clinical, and systemic factors. This heterogeneity in data presents a critical barrier to effective and equitable model training in federated settings [39].

In federated public health systems, data heterogeneity arises from numerous sources:

- Demographic variation: Different regions exhibit varying age distributions, socioeconomic statuses, comorbidity profiles, and health behaviors [66].
- Clinical practice patterns: Institutions may differ in diagnostic criteria, treatment protocols, and reporting standards [67].
- Technological infrastructure: Variations in electronic health record (EHR) systems, data granularity, and missingness rates [68].
- Geographical and environmental factors: Local climate, pollution levels, population density, and access to care impact disease patterns [44].

As a result, each data node contributes updates derived from distinct data distributions, which often leads to conflicts during global model aggregation reducing model convergence speed, stability, and accuracy.

The impact of non-IID data manifests in several key areas:

- Model Divergence: Local models trained on vastly different distributions may produce gradients that cancel each other during aggregation, reducing learning efficiency [65].
- Bias and Inaccuracy: The global model may disproportionately reflect data from dominant or well-resourced institutions, neglecting minority or rural populations [6].
- Reduced Generalizability: If the aggregated model is skewed toward specific cohorts, its performance across the entire population diminishes specially for subgroups not well represented in training data [44].
- Overfitting Local Trends: Local updates may overfit regional idiosyncrasies, hindering the model’s ability to extract general patterns across nodes [66].

Several studies included in this review encountered performance degradation due to non-IID data:

- A nationwide FL model for diabetes prediction showed significantly higher accuracy in urban hospitals than in rural clinics [64].
- In a COVID-19 surveillance system, local data from under-resourced regions had high missingness, resulting in poorly updated local models and limited global model contribution [44].
- Attempts to integrate behavioral data from mobile apps with hospital EHRs faced feature misalignment and inconsistent data semantics [39].

Researchers have proposed several technical approaches to address non-IID challenges:

- Personalized Federated Learning:Each node receives a customized model tailored to its local distribution, improving performance without fully sacrificing global generalization [69].
- Clustered Federated Learning: Nodes are grouped into clusters based on similarity, and separate models are trained per cluster before optionally aggregating [70].
- Data Harmonization and Preprocessing: Aligning data schemas and applying imputation or normalization techniques can reduce inter node variance [71].
- Adaptive Weighting: Contributions to the global model are weighted based on update reliability or data representativeness [72].
- Model Architecture Adaptation: Using modular or hierarchical models that can adapt parts of the architecture to local distributions while keeping shared layers globally synchronized [73].

For public health policymakers, the presence of non-IID data highlights the importance of:

- Supporting standardization of health data collection and coding practices [74];
- Investing in digital infrastructure to improve data quality in underrepresented regions [39];
- Establishing fairness metrics for evaluating FL-based health policy tools [6];
- Encouraging transparency and documentation in FL model reporting [71].

Without addressing non-IID data issues, there is a risk that federated public health models may perpetuate existing inequalities or produce ineffective interventions for vulnerable populations.

Table 5 outlines the key limitations of FL in public health and the corresponding mitigation strategies. Challenges such as non-IID data, schema heterogeneity, resource imbalance, and privacy risks are addressed through algorithmic, preprocessing, and security-enhancing methods. Additional issues including explainability, fairness, and regulatory barriers highlight the need for tailored frameworks to ensure reliable, equitable, and scalable FL adoption in healthcare.

#### 3.3.1. Resource Constraints and Technical Complexity

While FL holds immense promise for privacy-preserving, equitable, and decentralized health data analysis, its real-world implementation in public health settings faces substantial resource constraints and technical complexity [75]. These barriers are specially pronounced in low- and middle-income countries (LMICs), rural areas, and under-resourced health institutions where technical capacity, digital infrastructure, and skilled personnel may be limited. These constraints not only affect the viability of deploying FL systems but also raise concerns about fairness, inclusion, and the sustainability of AI-based health policy interventions [64].

FL requires each participating node to locally store data, execute model computations, and securely communicate model updates. However, many institutions, particularly in LMICs, struggle with the following:

- Limited access to reliable internet connectivity [76].
- Outdated or insufficient computing hardware (CPUs/GPUs) [77].
- Lack of robust cybersecurity frameworks [71].
- Poor digitization of health records or fragmented EHR systems [78].

As a result, the computational burden and communication demands of FL can exclude institutions that lack minimum technical requirements, thereby reinforcing existing inequalities in data-driven health decision-making.

Deploying and maintaining FL systems requires highly skilled personnel with expertise in the following:

- ML and deep learning [79].
- Secure multi-party computation and differential privacy [80].
- Federated optimization algorithms [65].
- Health informatics and data integration [39].

In many public health systems, particularly at the regional or community level, such interdisciplinary technical capacity is scarce. Without targeted training and institutional support, FL pilots may fail to scale or yield unreliable outputs. Furthermore, collaboration between computer scientists and health policymakers remains limited, making the co-design of federated systems challenging.

Beyond infrastructure and skills, FL deployments are often hindered by logistical complexity, including the following:

- Synchronization of updates across nodes with different computing speeds or schedules [63].
- Standardization of data schemas across diverse EHRs [68].
- Establishing secure communication protocols and legal agreements between participating institutions [71].
- Monitoring model performance and error propagation across the network [67].

These operational demands increase the cost and time required for implementation, which may deter health authorities from investing in FL-based tools, particularly when compared to more conventional centralized methods.

Recent studies have raised concerns about the energy demands of decentralized training frameworks. Unlike centralized models that train once on a pooled dataset, FL involves repeated training at each node and multiple rounds of aggregation. When deployed across large public health networks, this process can significantly increase the carbon footprint of AI-based health infrastructure particularly if training is unoptimized or runs on non-green energy sources [64,81].

This environmental consideration adds another layer of complexity for public health systems seeking to balance technological innovation with sustainability goals.

If left unaddressed, resource constraints and technical barriers can result in a two tiered public health AI ecosystem where only well-funded, digitally mature institutions benefit from federated models, while low-resource actors are left behind. This undermines the central promise of FL: inclusive, equitable, and privacy-preserving health data collaboration.

To mitigate these risks, policy recommendations include the following:

- Investment in digital health infrastructure and capacity-building [75].
- Development of lightweight and energy-efficient FL algorithms [64].
- Creation of national FL frameworks tailored to public sector constraints [39].
- Public–private partnerships to support open-source FL tools and decentralized platforms [71].
- Incentivizing collaborative networks that include rural and low-resource participants [80].

#### 3.3.2. Governance, Ethics, and Regulatory Barriers

FL, by design, offers a decentralized alternative to traditional AI systems by allowing model training on local data without raw data exchange. While this approach aligns with privacy-preserving goals, it introduces a new layer of complexity in governance, ethics, and regulation, particularly in the highly sensitive and heterogeneous domain of public health. For FL to be a viable tool in policy-driven disease prevention, it must operate within complex legal, institutional, and ethical frameworks that often vary across regions and nations [5,57].

One of the most significant barriers to FL in public health is the absence of standardized global or national regulatory frameworks explicitly addressing FL. Key legal concerns include the following:

- Unclear liability in cases of model failure or misdiagnosis due to federated updates [82].
- Data ownership ambiguity even though raw data is not shared; model updates may still leak information and trigger jurisdictional disputes [57].
- Regulatory gaps in defining what constitutes “data sharing” under laws like the GDPR, HIPAA (USA), or India’s Data Protection Bill [83].

Since FL operates on encrypted model gradients rather than raw data, existing regulations may either over-restrict its use due to lack of clarity or permit loopholes that pose new risks. This regulatory uncertainty discourages many health institutions from adopting FL at scale.

In large-scale FL networks, especially those spanning multiple countries or jurisdictions, differences in governance models pose substantial coordination challenges. Issues include the following:

- Inconsistent institutional policies on data security, retention, and usage rights [84].
- Lack of unified oversight bodies to ensure ethical model deployment and compliance monitoring [57].
- Fragmentation of legal accountability, especially when federated models are deployed in real-time for outbreak detection or policy support [6].

Despite its privacy-enhancing design, FL is not ethically exempt. Important ethical concerns include the following:

- Informed consent: Many FL deployments are built atop EHRs or registries where patients have not explicitly consented to their data being used for decentralized AI training [85].
- Fairness and representation: FL networks that exclude low-resource regions due to lack of infrastructure inadvertently introduce bias, resulting in policies that overlook marginalized populations [86].
- Opacity of model decisions: The decentralized and encrypted nature of FL complicates auditing and explaining model behavior, reducing stakeholder trust in public health contexts [39].

Unlike traditional AI systems, FL models may evolve continuously as they receive new updates from distributed sites. This dynamic nature complicates ethical review board (IRB) approvals, which are typically based on static protocols. Challenges include the following:

- How should continuous model updates be handled post-approval? [71]
- Should IRBs require access to model performance logs or node metadata? [82]
- How should consent withdrawal be managed when patient-level data are never transferred but still influence model parameters? [87]

These questions remain open and call for new ethical review frameworks tailored to FL.

To address these governance, ethics, and legal issues, the following steps are recommended:

- Develop FL-specific data governance frameworks involving regulators, ethicists, clinicians, and technologists [57].
- Adopt standardized legal templates for inter-institutional FL collaborations that clarify data rights and model liability and update ownership [88].
- Establish regulatory sandboxes to test FL models under controlled conditions while evolving compliance policies [75].
- Implement ethical auditing protocols that include fairness checks, bias audits, and explainability tools for FL systems [6].
- Incorporate federated IRB models to oversee multi-institutional FL research ethically and efficiently [30].

## 4. Discussion

### 4.1. Principal Findings and Interpretation

This systematic review critically examined the current landscape of FL applications in public health, emphasizing the role of FL in enabling equitable, privacy-preserving, and decentralized disease prevention policies. The synthesis of included studies revealed a rapidly expanding interest in FL as a viable alternative to centralized AI models, particularly in response to growing concerns over data sovereignty, privacy, and representation in global health systems.

Our findings on privacy-preserving cross-site modeling and improved generalizability are consistent with recent reviews of healthcare FL, which also emphasize limited real-world deployments and persistent heterogeneity. Unlike method-centric surveys, our synthesis foregrounds public health functions surveillance, risk stratification, policy support and governance issues, fairness auditing, legal contracting, and capacity gaps. This extends prior work by connecting FL methods to policy-level needs like standards, regulatory sandboxes, procurement templates, and equity like fair performance across sites and populations [6,89].

#### 4.1.1. Summary of Key Findings

1. Adoption Across Disease Domains: FL is being actively explored in both infectious disease control and chronic disease management, diabetes risk prediction, and cancer stratification. Its ability to integrate geographically and demographically dispersed datasets without direct data exchange has made it particularly attractive in multi-jurisdictional public health collaborations [44,67].
2. Predominance of Horizontal FL Architectures: Most reviewed studies implemented horizontal FL where datasets across institutions share the same features but differ in patient samples. Vertical FL and hybrid architectures were underrepresented, highlighting a current research gap in modeling across heterogeneous feature spaces [65].
3. Opportunities Identified: FL presents several high-value opportunities in public health policy:
  - Early warning systems using distributed real-time data;
  - Predictive analytics tailored to local epidemiology;
  - Risk stratification models that preserve population diversity;
  - Support for data sovereignty, enabling regions to retain control over their data while contributing to global models.
4. Challenges Reported: Despite these advantages [39], critical barriers remain:
  - Non-IID data significantly hamper model performance and generalizability;
  - Infrastructure disparity limits equitable participation from low-resource regions;
  - Ethical and regulatory ambiguity undermines confidence in implementation;
  - Lack of model interpretability and fairness auditing, especially in policy-sensitive domains like vaccine distribution.
5. Underrepresentation of LMICs: The majority of FL deployments and studies were conducted in high income countries or technologically advanced institutions. This reveals a substantial equity gap and calls for urgent attention to supporting FL readiness in underrepresented regions [64].

#### 4.1.2. Interpretation of Findings in Public Health Context

The findings of this review suggest that FL is more than a technical innovation; it represents a structural shift in how public health data can be ethically leveraged. By minimizing centralized data pooling, FL aligns with modern demands for privacy, patient agency, and cross-border data governance. However, these benefits are currently being realized in siloed or well-funded networks, rather than in comprehensive global health systems.

Moreover, the observed trade-offs such as accuracy vs. fairness, or efficiency vs. inclusivity highlight the need for context-aware policy integration. In outbreak settings, FL can enhance response agility without breaching patient confidentiality. In chronic care, it can help build longitudinal models across dispersed populations. Yet, such applications must be co-designed with public health stakeholders to ensure that model objectives, thresholds, and outputs serve diverse health equity goals.

### 4.2. Comparison with Centralized AI Models

FL and centralized AI models represent two fundamentally different paradigms for data-driven health analytics. While both aim to extract meaningful insights from health data to support disease prevention, policymaking, and clinical decision support, they differ substantially in terms of data architecture, ethical alignment, scalability, inclusivity, and operational feasibility. This subsection provides a detailed comparison between FL and centralized AI, specifically in the context of public health applications.

#### 4.2.1. Data Governance and Privacy

Centralized AI models rely on the aggregation of raw data into a single server or cloud repository for model training. This architecture presents a well-known privacy risk, especially in public health domains that handle sensitive personal, demographic, and behavioral data [90].

In contrast, FL keeps data localized at the point of generation, transferring only encrypted model updates. This structure significantly reduces the risk of data breaches, supports compliance with data protection laws such as GDPR and HIPAA, and enhances trust among stakeholders [52].

Table 6 compares centralized AI and FL with respect to data privacy. While centralized AI requires raw data transfer and faces high privacy risks, FL mitigates these concerns by keeping data local and only sharing model updates. This design not only lowers privacy risks but also makes FL more adaptable to legal and regulatory requirements across jurisdictions.

#### 4.2.2. Equity and Inclusivity

Centralized AI models often marginalize low-resource or underrepresented institutions that cannot share data due to infrastructure or regulatory barriers. This exclusion results in biased models that perform poorly in diverse or vulnerable populations [91].

#### 4.2.3. Technical and Infrastructural Requirements

Centralized models benefit from simplicity and speed in computation due to the presence of all data in one location. However, they require secure transmission channels and massive storage, which may not scale easily.

FL, while more complex operationally, scales better in geographically distributed environments, especially when aligned with cloud edge computing systems. However, FL is sensitive to the following:

- Non-IID data across institutions;
- Computational disparities (e.g., slow clients, weak hardware);
- Communication delays in synchronous updates.

Thus, FL requires coordination protocols, model personalization techniques, and aggregation algorithms, FedAv and FedProx, to be effective.

#### 4.2.4. Adaptability in Emergency and Outbreak Scenarios

During pandemics like COVID-19, centralized AI systems were slow to deploy globally due to bottlenecks in data access and compliance. FL allows rapid local training and global aggregation, enabling real-time predictive analytics across countries or health regions without pooling sensitive patient data [21,89,92].

This makes FL particularly suitable for the following:

- Outbreak detection;
- Syndromic surveillance;
- Border-crossing epidemiological collaboration.

#### 4.2.5. Interpretability and Transparency

Centralized models allow easier explainability audits, as developers have full access to all data and model outputs. In FL, since data remain distributed and often encrypted, interpretability becomes more difficult. However, recent developments are closing this gap [93,94].

Ethically, both paradigms must ensure transparency in model reasoning, but FL requires additional tools and frameworks to audit model behavior remotely.

#### 4.2.6. Cost and Sustainability

Centralized models often centralize both computation and cost, potentially becoming financially unsustainable for public sector applications in LMICs.

FL distributes computation across nodes, potentially reducing bottlenecks but increasing energy consumption and infrastructure costs at the edge. When optimized, FL can offer a sustainable model, particularly in settings with fragmented health infrastructure [5,95].

Table 7 provides a comprehensive comparison of centralized AI and FL in public health applications. The table illustrates how FL offers advantages in privacy, inclusivity, scalability, and compliance with legal frameworks, while centralized AI may excel in accuracy and explainability when large, diverse datasets are centrally available. However, each paradigm faces distinct challenges: Centralized AI struggles with privacy risks and regulatory barriers, whereas FL must overcome issues of statistical heterogeneity, local infrastructure capacity, and limited transparency. Together, these trade-offs highlight the complementary roles of centralized and federated approaches in advancing trustworthy AI for public health.

### 4.3. Policy Implications and Framework Alignment

The integration of FL into public health decision-making not only introduces technical innovation but also necessitates a transformation in how policies are conceptualized, enacted, and regulated. The implications of FL extend beyond ML infrastructure; they influence global health equity, data protection, governance models, and cross-sectoral collaboration. This subsection unpacks the key policy implications of FL adoption and examines its alignment with existing health and data governance frameworks [38].

#### 4.3.1. Enabling Privacy-by-Design in Public Health Systems

FL inherently aligns with the “privacy-by-design” principle, a foundational component of regulations such as the General Data Protection Regulation (GDPR), HIPAA, and India’s Digital Personal Data Protection Act. By minimizing data transfer and ensuring sensitive health information never leaves local environments, FL supports public health systems in maintaining patient confidentiality while still engaging in data-driven policymaking [39].

Policy implication: Governments and health agencies can integrate FL into national eHealth and digital surveillance frameworks without renegotiating data sovereignty agreements [38].

#### 4.3.2. Promoting Equitable Participation in Global Health Initiatives

Conventional AI systems disproportionately represent data from technologically advanced nations and urban centers. FL enables institutions in low- and middle-income countries (LMICs) to contribute to global disease models without needing to export raw data, a practice often restricted due to legal or infrastructural constraints [95].

Policy implication: Federated architectures can be embedded into WHO’s Health Data Governance framework and SDG-aligned national health strategies, enhancing inclusivity and fairness [57].

#### 4.3.3. Strengthening Cross-Border Collaboration in Epidemic Intelligence

FL is particularly valuable in cross-border disease surveillance, where countries may hesitate to share data due to political or legal concerns. By allowing joint model training without centralized data collection, FL reduces friction in multilateral collaborations.

Policy implication: FL aligns with the International Health Regulations (IHR 2005), supporting real-time risk analysis while respecting national data laws [102].

#### 4.3.4. Adapting Legal Frameworks and Procurement Policies

To fully adopt FL, governments may need to update the following:

- Health IT procurement guidelines to include edge-computing requirements;
- Cybersecurity and medical device laws to support decentralized learning;
- Data residency laws to recognize encrypted parameter exchange as compliant.

Policy implication: Legislative modernization is essential to mainstream FL in digital health programs such as national disease registries, telemedicine networks, and real-time biosurveillance platforms [23].

#### 4.3.5. Establishing Public–Private Partnerships

Implementing FL at scale often requires collaboration across ministries of health, AI vendors, telecom providers, and academic institutions. Governments can commit to the following:

- Incentivize private sector FL tools aligned with public objectives;
- Use regulatory sandboxes to test FL for health applications;
- Create FL consortia under existing health innovation programs.

Policy implication: Public-private partnerships will be critical to scaling FL for public health, specially in areas requiring interoperability and infrastructure co development [20].

### 4.4. Addressing Data and Infrastructure Inequality

Despite its privacy-preserving and collaborative advantages, the widespread adoption of FL in public health faces a critical barrier: inequality in data quality and digital infrastructure across participating institutions and regions [103]. These disparities risk perpetuating or even widening existing inequities in public health outcomes, particularly when under-resourced communities are underrepresented in model development. This subsection examines the multifaceted nature of data and infrastructure inequality and outlines strategies to address it within the FL paradigm [92].

**Figure 3:**
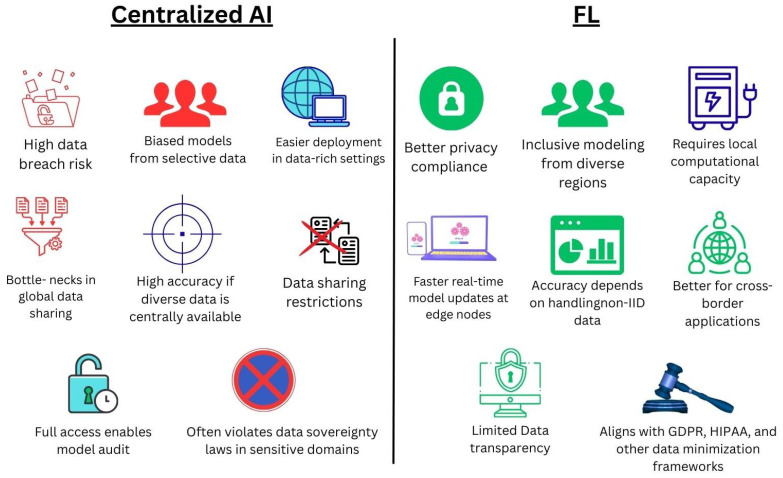
Comparison of centralized AI and federated learning in public health applications.

Figure 3 compares centralized AI and FL in terms of their strengths and limitations for public health applications. Centralized AI often achieves high accuracy when diverse datasets are pooled but faces significant risks like privacy breaches, biased modeling, data-sharing bottlenecks, and violations of data sovereignty laws. In contrast, FL enhances privacy compliance, supports inclusive modeling across diverse regions, and enables faster real-time updates at the edge, though it requires sufficient local computational capacity and careful handling of non-IID data.

#### 4.4.1. Unequal Access to Digital Infrastructure

FL requires basic technical infrastructure such as internet connectivity, computing hardware, secure storage, and encryption protocols. While high-income countries (HICs) and major academic health centers often have such systems in place, many low- and middle-income countries (LMICs) and rural or community-based clinics face the following constraints:

- Limited broadband access and unreliable electricity supply;
- Outdated or no local computing hardware;
- Inadequate cybersecurity frameworks;
- Shortage of trained IT and data personnel.

These limitations impede the ability of many regions to participate fully in federated networks, resulting in model training skewed toward digitally rich environments [22].

#### 4.4.2. Disparities in Data Quantity and Quality

In federated systems, each client (hospital, region, or node) contributes to model updates using local data. However, there are substantial disparities in the following areas:

- Data volume: Smaller institutions may have insufficient case numbers.
- Data completeness: Underfunded systems often contain missing or low-quality data.
- Semantic heterogeneity: Inconsistent labeling or coding across regions.
- Bias and underrepresentation: Marginalized groups may be absent from datasets.

As a result, federated models may overfit to dominant nodes and underperform for minority or low-data clients, ironically reinforcing health inequalities [104].

#### 4.4.3. Algorithmic and Architectural Strategies for Mitigation

To counteract inequality in FL participation and performance, several techniques can be integrated:

- Weighted Aggregation, FedAvgM and FedProx: Adjusts influence based on data quality or size.
- Personalized FL: Allows local fine-tuning for contextual relevance.
- Data Augmentation at the Edge: Synthetic data generation for low sample clients.
- Knowledge Distillation and Transfer Learning: Share global insights with weaker nodes.
- Edge Resource Optimization: Deploy lightweight models using efficient runtimes (e.g., TensorFlow Lite).

These approaches allow even resource-constrained nodes to participate and benefit from federated networks.

#### 4.4.4. Policy and Investment Considerations

Mitigating inequality in FL adoption is not just a technical challenge; it requires targeted policy interventions and global investment:

- Funding schemes for FL infrastructure in LMICs.
- Digital literacy and workforce training programs.
- Inclusion of FL in health system strengthening by donors.
- National data standardization for interoperability.

Without these support structures, FL risks reinforcing rather than reducing digital health inequities.

### 4.5. Ethical and Legal Considerations in FL Deployment

As FL gains traction in public health research and disease prevention policy, its implementation must be guided by robust ethical and legal frameworks. Unlike traditional centralized ML approaches, FL decentralizes both data and computation, introducing unique challenges and responsibilities regarding individual rights, institutional accountability, algorithmic fairness, and regulatory compliance.

#### 4.5.1. Respect for Autonomy and Informed Consent

In public health, data subjects often do not provide individual consent for every analytical use of their data, especially when aggregated into registries or surveillance databases [57,105,106]. FL, while reducing privacy risk, still engages personal health data indirectly via local computations.

Key concerns include the following:

- Whether data subjects can meaningfully opt in or opt out of FL participation;
- If existing consent agreements cover federated model training;
- How dynamic consent models can be integrated into federated architectures.

FL systems should perform the following:

- Provide transparent documentation on data usage;
- Support institutional and community-level governance;
- Explore user-controlled data access and federated consent mechanisms.

#### 4.5.2. Privacy, Confidentiality, and Data Minimization

Although FL avoids centralizing data, it does not eliminate privacy risks [107]. These include the following:

- Model inversion attacks;
- Gradient leakage of sensitive attributes;
- Reduced visibility into system-wide data misuse or bias.

Best practices include the following:

- Differential privacy;
- Secure multi-party computation and encryption protocols;
- Regular third-party audits and adversarial robustness testing.

These align with laws such as GDPR (EU), HIPAA (USA), and India’s Digital Personal Data Protection Act (DPDPA).

#### 4.5.3. Algorithmic Fairness and Non-Discrimination

FL may inadvertently reinforce bias if training favors data-rich institutions [108,109]. This can result in the following:

- Skewed disease predictions across demographic or geographic groups;
- Unequal allocation of health resources.

Solutions include the following:

- Weighted aggregation to account for diversity;
- Performance auditing across subgroups;
- Use of fairness-aware FL methods (e.g., FairFed, FedAUX).

These steps align with ethical principles of distributive justice and non-discrimination.

#### 4.5.4. Accountability and Transparency

The distributed nature of FL complicates the following:

- Traceability of model errors;
- Assignment of responsibility;
- Post hoc explainability and legal liability [110].

To address this, institutions should carry out the following:

- Log participation, updates, and training metrics;
- Use federated explainability tools (e.g., SHAP, LIME);
- Formalize roles and responsibilities via contracts or MoUs.

#### 4.5.5. Regulatory and Jurisdictional Challenges

FL operates across borders, challenging conventional legal frameworks due to the following:

- Conflicting data residency laws;
- Ambiguous classification of model updates as data processing;
- Lack of unified AI regulation for decentralized systems [111].

Policy solutions include the following:

- Creating FL-specific compliance toolkits;
- Establishing AI regulatory sandboxes;
- Supporting cross-border FL consortia and standards.

### 4.6. Limitations of the Review

Despite offering valuable insights into the potential and challenges of FL in public health policy, this systematic review is not without limitations. Acknowledging these constraints is essential for transparency, interpretation, and guiding future research. Below, we present a detailed discussion of the methodological, thematic, and contextual limitations.

Table 8 summarizes the main limitations of this systematic review. The evidence base remains limited, with only a few empirical FL studies applied to public health, while heterogeneity across architectures, disease domains, and metrics hinders synthesis. Exclusion of non-English and gray literature introduces publication bias, and the absence of meta-analysis prevents pooled statistical assessment. Additionally, the rapid evolution of FL means recent advances may not be captured, and some reviewer subjectivity may have influenced thematic categorization. These limitations should be considered when interpreting the findings and recommendations.

#### 4.6.1. Limited Number of Empirical Studies in Public Health

While FL has seen growing application in clinical and biomedical domains, its adoption in public health, especially at the policy level, remains nascent [112]. This constrained the pool of eligible studies and resulted in the following:

- Limited our ability to conduct meta-analysis or robust subgroup comparisons;
- Required inclusion of proof-of-concept or pilot studies;
- May have led to an overrepresentation of institutional healthcare settings versus national or population-based policy implementations.

The synthesis reflects an emerging research area rather than a fully established body of evidence.

#### 4.6.2. Heterogeneity in Study Designs and Reporting

Included studies varied widely in the following areas:

- Application domains (e.g., communicable vs. non-communicable diseases);
- FL architectures (horizontal, vertical, or hybrid);
- Outcome metrics and evaluation criteria.

This heterogeneity restricted direct comparison and limited generalizability due to inconsistent reporting of datasets, validation metrics, and privacy-preserving mechanisms [113].

#### 4.6.3. Language and Publication Bias

We limited inclusion to peer-reviewed articles in English from selected databases. The consequences are as follows:

- Non-English studies and gray literature (e.g., NGO reports, government documents) were excluded;
- FL applications in LMICs and low-resource settings may have been underrepresented.

The findings may reflect high-resource, English-speaking contexts disproportionately [114].

#### 4.6.4. Absence of Quantitative Meta-Analysis

Due to the qualitative nature of most studies and variability in reported metrics, no quantitative meta-analysis was performed. While thematic synthesis and tabular summaries were provided, the lack of pooled statistical estimates limits definitive conclusions on performance benchmarks [52].

#### 4.6.5. Timeframe Constraints and Rapid Field Evolution

The review was completed over a defined window (2020–2025), during which FL technology and frameworks are rapidly evolving. Newer publications with post-search cutoffs may provide updated methods, case studies, or regulatory guidance.

#### 4.6.6. Potential Reviewer Subjectivity

Despite the use of structured tools (e.g., PRISMA, quality assessment checklists), reviewer bias could influence the following:

- Interpretation of FL implementations;
- Categorization of disease domains and outcomes;
- Inclusion/exclusion decisions during full-text screening.

Cross-validation by multiple authors helped reduce subjectivity [115].

### 4.7. Future Directions

While FL offers transformative potential in the realm of privacy-preserving public health intelligence, its practical deployment across diverse health systems remains in early developmental stages. Building on the current landscape of opportunities and challenges outlined in this review, several strategic directions are recommended for advancing research, development, and policy implementation of FL in disease prevention.

**Figure 4:**
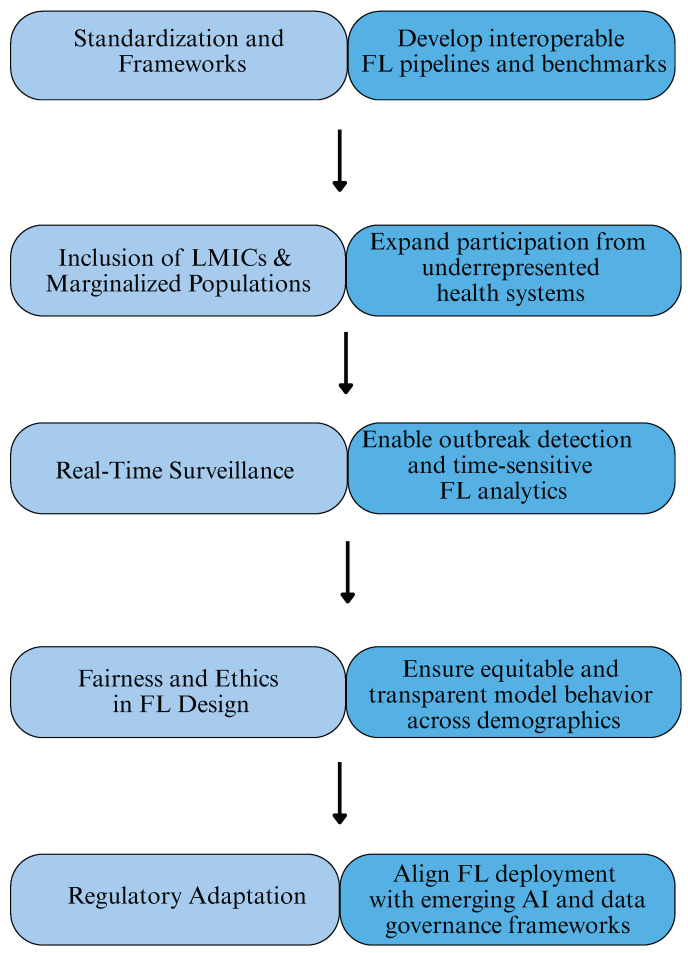
Future directions roadmap for FL in public health. The roadmap shows key thematic areas including standardization, inclusion, surveillance, fairness, regulatory adaptation, and capacity-building aligned with progressive stages of implementation and policy integration.

Figure 4 illustrates a roadmap for advancing FL in public health, emphasizing standardization, inclusivity, surveillance, fairness, and regulatory adaptation. It underscores the importance of building interoperable frameworks, engaging underrepresented health systems, enabling timely outbreak detection, ensuring equity and transparency in model design, and aligning with emerging governance structures. This progression highlights how FL can be integrated into policy and practice while addressing technical, ethical, and structural challenges.

#### 4.7.1. Development of Standardized FL Frameworks and Protocols

To facilitate interoperability and reproducibility across public health institutions, there is a pressing need for the following:

- Unified FL pipelines: Including reference architectures, data schemas, and secure aggregation protocols tailored for public health datasets (e.g., surveillance, EHRs, immunization records).
- Benchmark datasets and evaluation metrics: Publicly accessible FL ready datasets and fairness-aware benchmarks for assessing model robustness across diverse population groups.
- Modular open-source platforms: Frameworks such as TensorFlow Federated and Flower should be extended with plug-ins for health specific model evaluation, fairness metrics, and privacy risk audits.

#### 4.7.2. Inclusion of Underrepresented and Low-Resource Settings

To ensure equitable AI model development, future FL research must include nodes from low- and middle-income countries (LMICs), rural health centers, and marginalized populations. Priorities include the following:

- Edge device optimization: Lightweight FL algorithms suitable for deployment in bandwidth-constrained environments.
- Synthetic data generation: Enabling augmentation of sparse datasets to improve training stability.
- Policy co-design with local stakeholders: Incorporating local health priorities and data stewardship norms into FL objectives and reward structures.

#### 4.7.3. Federated Learning for Real-Time Disease Surveillance

There is an emerging opportunity to use FL for continuous, decentralized outbreak detection and response [116]. Future initiatives should focus on the following:

- Time series FL models: Capable of adapting in real-time to changing epidemiological patterns.
- Cross jurisdictional coordination platforms: Secure multinode governance models for international epidemic intelligence.
- Integration with digital contact tracing and syndromic surveillance tools: To enhance early warning systems while preserving data sovereignty.

#### 4.7.4. Federated Fairness and Ethical Algorithm Design

The next generation of FL systems must explicitly address fairness, accountability, and transparency:

- Personalized FL and subgroup modeling: Algorithms that maintain both global performance and subgroup-specific accuracy.
- Federated explainability: Development of model interpretation tools compatible with decentralized architectures.
- Ethical auditing frameworks: Independent oversight of FL system impact on population level health equity.

#### 4.7.5. Adaptive Regulatory and Legal Frameworks

Governments and health authorities should work toward enabling governance environments that facilitate ethical FL deployment [26]:

- Federated data protection standards: Legal definitions for encrypted gradient sharing, local processing accountability, and data fiduciary responsibilities.
- AI-specific public health guidance: Harmonized FL regulations within broader digital health strategies.
- Regulatory sandboxes: Allowing experimentation with FL applications in controlled, low-risk settings before full-scale implementation.

#### 4.7.6. Capacity Building and Workforce Training

For federated systems to be sustainable [44], investment is needed in human capital:

- Training public health professionals: In FL concepts, privacy engineering, and ethical AI deployment.
- Building cross disciplinary teams: Integrating epidemiologists, data scientists, ethicists, and legal experts.
- Promoting global south participation: Through fellowships, open-source contributions, and institutional partnerships.

### 4.8. Policy Recommendations

1. Adopt FL pilots for priority use-cases surveillance, early warning, and risk stratification with clear success metrics like timeliness, accuracy, and fairness.
2. Publish procurement and governance templates like data-processing agreements, model update contracts, and audit clauses compatible with GDPR/HIPAA and cross-border deployments.
3. Standardize data and metadata using open schemas and controlled vocabularies to enable cross site learning without custom plumbing.
4. Mandate privacy by design, such as secure aggregation; implement differential privacy where feasible and report fairness metrics by region, facility type, and population group.
5. Invest in capacity-building computing, networking, MLOps, and workforce training to include low-resource settings, reducing bias and improving generalizability.

## 5. Conclusions

This systematic review examined the emerging role of FL as a decentralized AI approach for advancing secure, equitable, and privacy-preserving disease prevention policies in public health. By synthesizing findings from a diverse range of studies, we highlighted how FL enables collaborative model development across institutions and jurisdictions without requiring the sharing of raw health data. This approach directly addresses contemporary concerns about data sovereignty, regulatory compliance, and ethical governance.

The review identified substantial opportunities offered by FL, including its application in early outbreak detection, predictive modeling, and risk stratification. Moreover, FL can contribute to more inclusive and fair public health policy design by incorporating data from underrepresented populations and preserving local autonomy over sensitive health information. These strengths are increasingly relevant in global health systems striving to balance innovation with ethical and legal accountability.

Nonetheless, significant challenges remain. Technical barriers such as non-IID data distribution, communication overhead, and limited digital infrastructure in low-resource settings must be addressed. Ethical concerns ranging from informed consent and fairness to explainability and liability also necessitate comprehensive legal and institutional frameworks. In addition, disparities in research coverage, reporting standards, and the absence of meta-analytical benchmarks hinder robust evidence synthesis and large-scale policy translation.

Looking forward, the successful integration of FL into public health infrastructure requires multi-sectoral collaboration. This includes standardizing FL protocols, investing in LMIC-ready technology, promoting inclusive algorithmic design, and fostering adaptive regulatory ecosystems. A parallel emphasis on capacity building, community engagement, and ethical AI governance is critical to ensuring that the benefits of FL are equitably distributed.

In conclusion, FL represents a promising paradigm shift toward decentralized intelligence in public health. When thoughtfully implemented, FL can catalyze the development of data-driven, context sensitive, and ethically aligned disease prevention policies, ultimately strengthening the resilience, fairness, and trustworthiness of global health systems.

## Figures and Tables

**Table 1:** Unified comparison of related work and the literature gaps addressed in public health federated learning (FL).

Scope	Methodology	Focus Area	Target Health System Level	Identified Gap	AI Methodology Challenge	Disease Focus	Reference
FL in public health and disease prevention	Systematic review (PRISMA)	Policy design, equity, ethical FL governance	National and cross-border public health systems	Lack of synthesis for policy; cross-border frameworks; fairness; national integration; benchmarking	Heterogeneity-robust FL; secure aggregation; standards	Both	*This Review (2025)*
FL in smart healthcare networks	Scoping review	Wearables, IoT in hospital environments	Institutional				[25]
FL for COVID-19 detection	Empirical study	Mobile symptom data, federated analysis	Regional healthcare systems			Infectious	[26]
Federated analytics for disease forecasting	Prototype framework	Pandemic prediction and response	Regional/ national public health agencies	Need for cross-border, privacy-preserving forecasting frameworks	Interoperability; model update security	Infectious	[27,28]
Ethical and regulatory aspects of FL	Conceptual review	Consent, fairness, explainability	Cross-level (individual to national)	Limited fairness/ethics integration in deployment	Fairness-aware training and auditing	Both	[21]
FL performance for chronic disease care	Experimental pipeline	Diabetes/CVD with EHRs	Institutional (hospital networks)	Underrepre sentation of chronic disease prevention	Longitudinal/ behavioral FL design	Chronic	[29]
System level optimization of FL	Technical survey	Communication efficiency, compression	Cross-domain	Underrepre sentation of chronic prevention; efficiency constraints	Communication-efficient, non-IID FL	Both	[5]
FL in LMIC rural settings	Field-based framework	Low-resource deployment	Regional clinics, rural LMICs	Limited evaluation in low-resource settings	Stable training under non-IID + weak networks	Infectious	[30]
FL for public health synthesis	Review/note	Policy-oriented evidence synthesis	Policy bodies	Lack of synthesis on FL for policymaking	Generalizability across heterogeneous data	Both	[6]
Cross-border data sharing for surveillance	Perspective/ framework	International collaboration	National/regional cross-border	Minimal analysis of cross-border frameworks; weak national integration	Interoperability; jurisdictional limits	Infectious	[31]
Fairness/bias in healthcare FL	Review/ analysis	Equity and bias mitigation	Cross-level	Limited ethical/fairness discourse in deployment	Bias propagation; subgroup performance gaps	Both	[32]
Benchmarking in health FL	Survey/ framework	Standards and evaluation	Cross-ecosystem	Lack of standardized benchmarking	Open metrics and datasets for public health FL	Both	[33]

**Table 2:** Summary of Iinclusion and exclusion criteria.

Criterion Category	Inclusion Criteria	Exclusion Criteria
Study Type	Peer-reviewed original research articles (experimental, observational, implementation, or modeling studies)	Editorials, preprints, white papers, commentaries, or conference abstracts without full peer review
Technology Focus	Studies applying or evaluating FL or similar decentralized AI approaches	Studies using only centralized AI models or unrelated ML techniques
Policy Relevance	Addressing public health decision-making, disease prevention, or population-level policy impact	Focused only on individual-level clinical tools with no public health or policy connection
Health Focus	Studies involving communicable or non-communicable diseases in human populations	Simulated, laboratory, or animal studies with no relevance to public health systems
Geographic Scope	Global scope, including low-, middle-, and high-income settings	Not applicable (no exclusions based on location)
Language	English only	Any language other than English
Publication Period	Published between 1 January 2020 and 30 June 2025	Published before 2020 or after June 2025

**Table 3:** Thematic categories emerging from the synthesis of included studies.

Synthesis Theme	Description	Representative Examples
Opportunities for FL in Disease Prevention Policy	Highlights the technical and operational advantages of FL in public health, such as secure data sharing, real-time collaboration, and modeling across diverse populations.	Early COVID-19 outbreak detection across hospitals; FL models for diabetes risk prediction using distributed primary care data.
Challenges to FL Deployment in Public Health	Documents technical, ethical, and infrastructural barriers including data heterogeneity, interoperability issues, governance gaps, and unequal access to computing resources.	Non-IID data across regions; lack of FL support in low-resource settings; absence of regulatory frameworks for decentralized AI.
Policy and System-Level Implications	Explores how FL contributes to health equity, privacy preservation, and local policymaking autonomy, while emphasizing the need for cross-sector collaboration.	Privacy-preserving modeling in Europe under GDPR; use of FL to guide local vaccination resource planning; partnerships between ministries of health and tech firms.

**Table 4:** Summary of federated learning architecture types across included studies.

FL Architecture Type	No. of Studies (n = 19)	Percentage (%)	Key Applications	Representative Use Cases
Horizontal Federated Learning (HFL)	11	57.9%	Epidemic modeling, chronic disease screening, multi-hospital EHR aggregation	COVID-19 prediction across provinces; diabetes screening via regional health centers
Vertical Federated Learning (VFL)	4	21.1%	Socio-clinical integration, cross-sector modeling, personalized risk assessment	Merging insurance and hospital data; combining wearables with EHRs for cancer risk
Hybrid/Customized Architectures	3	15.8%	Smart surveillance, edge-to-cloud FL, hierarchical governance	FL with mobile apps and hospitals; city-level FL aggregation for vaccine allocation
Unspecified/Not Reported	1	5.2%	General policy modeling with no defined architecture	Public health early-warning model with unclear system structure

**Table 5:** Federated learning limitations and mitigation strategies in public health applications.

Limitation	Impact on Public Health Modeling	Mitigation Strategy	Method Type	Application Area	Reference
Non-IID Data Distribution	Model divergence; poor generalization across populations	Clustered FL; FedProx; personalization layers	Algorithmic	Disease risk prediction, surveillance	[65,66]
Data Schema Heterogeneity	Feature misalignment between nodes	Data harmonization; standard ontologies	Preprocessing	Multi-site EHR integration	[39,71]
Resource Imbalance Across Nodes	Slower nodes delay training; accuracy skewed by high-resource institutions	Adaptive model aggregation; asynchronous FL	Infrastructure-aware FL	Rural vs. urban health centers	[63]
Privacy Leakage from Gradients	Inference of sensitive data from model updates	Differential privacy; secure aggregation	Security-enhancing	Sensitive patient data	[5]
Lack of Model Explainability	Difficulty interpreting risk scores; mistrust by clinicians	Use of interpretable models; SHAP in FL	Explainable AI	Clinical decision support	[35]
Bias Toward Dominant Institutions	Unequal influence on global model	Fair weighting schemes; participation normalization	Fairness-aware FL	Multi-region model development	[26]
Slow Convergence in Heterogeneous Settings	Extended training time and communication costs	FedAvg optimization; learning rate tuning	Algorithm optimization	Pandemic-time model deployment	[72]

**Table 6:** Comparison of FL and centralized AI on data privacy.

Aspect	Centralized AI	Federated Learning
Raw Data Movement	Required	Not required
Privacy Risk	High	Low
Legal Compliance	Challenging in multi- jurisdiction settings	More compliant due to data locality

**Table 7:** Comprehensive comparison of centralized AI and federated learning in public health applications.

Aspect	Centralized AI	FL	Advantage	Challenges	Reference(s)
Data Privacy and Security	Requires raw data transfer to central server	Keeps data local and shares only encrypted model updates	FL, Better privacy compliance	Centralized AI— High breach risk	[68,71]
Equity and Representation	Often excludes under-resourced or data-restricted institutions	Allows wider participation regardless of location or resources	FL, Inclusive modeling from diverse regions	Centralized, Biased models from selective data	[95,96]
Infrastructure Dependence	Requires high-capacity central server	Distributed computing required across all clients	Centralized, Easier deployment in data-rich settings	FL, Requires local computational capacity	[63,65]
Speed of Deployment	Slower in multi-region scenarios due to regulatory friction	Faster real-time model updates at edge nodes	FL, Suited for outbreaks and emergency response	Centralized, Bottlenecks in global data sharing	[21,67]
Model Accuracy and Robustness	High accuracy if diverse data are centrally available	Accuracy depends on handling non-IID data	Centralized, When full data is accessible	FL, Requires optimization for statistical heterogeneity	[97,98]
Scalability	Limited scalability in international or multi-hospital projects	High scalability via federated nodes and asynchronous updates	FL, Better for cross-border applications	Centralized, Data sharing restrictions	[65,99]
Explainability	Easier due to full data access	Requires development of remote XAI tools	Centralized, Full access enables model audit	FL, Limited transparency	[100]
Legal and Ethical Governance	Often violates data sovereignty laws in sensitive domains	Aligns with GDPR, HIPAA, and other data minimization frameworks	FL, Privacy by design	Centralized, May breach consent frameworks	[39,101]

**Table 8:** Summary of limitations of the systematic review.

Limitation Category	Description	Implication
Limited Evidence Base	Few empirical FL studies applied directly to public health policy; inclusion of conceptual or pilot frameworks	Limits generalizability and robustness of conclusions
Study Heterogeneity	Wide variation in FL architecture, disease domains, and outcome metrics	Hinders direct comparison or synthesis
Language and Publication Bias	Exclusion of non-English, gray literature, and LMIC pilot programs	Skews findings toward English-speaking, high-income settings
No Meta-Analysis Conducted	Lack of standardized metrics across studies prevented pooled statistical analysis	Limits quantification of effect sizes and accuracy
Rapid Field Evolution	Review cutoff may exclude recent advances post-2024	Limits inclusion of latest tools, models, and policies
Reviewer Subjectivity	Some subjectivity in categorization, eligibility, and thematic coding	Potential influence on thematic synthesis
